# Neglected gaps in improving the health, wellbeing, and care for sexual and gender minority young people living in low- and lower-middle- income countries: a scoping review

**DOI:** 10.1186/s12889-023-16443-8

**Published:** 2023-08-30

**Authors:** Cara Frances, Camille Garnsey, Jessica DeMulder

**Affiliations:** 1 Population Council, New York, NY USA; 2 Department of Psychological Sciences, University of Connecticut, Storrs, CT USA

**Keywords:** Sexual and gender minority, LGBTQ, Youth, Adolescent health, Adolescent wellbeing, Scoping review, LMICs, Majority World

## Abstract

**Background:**

There is a lack of reliable data on the size, characteristics, and experiences of sexual and gender minority (SGM) young people (ages 10–24) in low- and lower-middle- income countries (LMICs). This review examines the research conducted in the last thirteen years with and about SGM young people living in low-income settings and seeks to answer the question: What is known about the mental and physical health needs, safety, and wellbeing of SGM young people living in LMICs?

**Methods:**

We conducted a scoping review informed by the methodological frameworks put forth by Arksey and O’Malley and the Joanna Briggs Institute. We systematically searched two general social science databases and one topic-specific database for peer-reviewed papers, of any research design, that included SGM young people or explored attitudes toward SGM young people in LMICs. We included papers that reported on factors influencing the health and wellbeing of SGM populations, including physical and mental health, healthcare-seeking behaviors, substance use, experiences of discrimination and/or stigma, experiences of violence and abuse (emotional, physical, and/or sexual), economically motivated paid sex practices, housing or economic security, and attitudes of others toward SGM populations.

**Results:**

Of the 5,409 unique records identified, 79 papers drawing from data collected from 74 unique studies met the inclusion criteria. Only 50 of the 79 papers included SGM young people as participants, with just 13 focusing exclusively on SGM young people ages 10–24. The included papers were classified into three thematic groupings: attitudes toward SGM populations (*n* = 26), risks to health (*n* = 40), and experiences of stigma and discrimination (*n* = 13).

**Conclusion:**

The findings indicate that the health and wellbeing of SGM young people in LMICs has been historically under-researched. While SGM young people have received more attention from researchers in recent years, the body of literature as a whole is disjointed and sparse, and often studies are *about* SGM young people, rather than with and for them. Our review highlights the need for more and better research, more accurate and disaggregated demographic data, and leadership and participation of SGM-led community-based organizations in the co-design of studies that focus on SGM young people.

## Background

Totaling 1.8 billion, today’s generation of young people (ages 10–24) are the the largest population of young people in history and nearly 60% live in low- and lower-middle- income countries (LMICs) [1]. The percentage of young people who identify as sexual and gender minorities (SGM)^1^ globally is unknown, because there is a lack of reliable data on the size of SGM populations [3]. Data from the United States, which often serves as the starting point for global models of SGM populations [3], suggests that more than 9.5% of adolescents ages 13–17 identify as SGM [4].

Evidence from high-income countries show that SGM young people are subjected to higher rates of peer victimization, stigmatization, social stress, and family and social rejection compared to their cisgender and heterosexual peers [5]. These experiences of discrimination, violence, and prejudice negatively impact the overall physical and mental health of SGM young people [6]. An emerging evidence base of meta-analyses and longitudinal studies in high-income countries have confirmed associations between these experiences and the development and maintenance of anxiety, depression, self-harm, and suicidal behavior, as well as poor immune and cardiovascular function [7–11]. In general, same-gender and same-sex sexual behavior and gender diverse expression is more aggressively sanctioned in LMICs than high-income countries: it is expressly criminalized in 41 LMICs, and punishable by death in seven [12]. In light of recent evidence from high-income countries linking the negative impacts of unsupportive environments (including legislative) to the health and wellbeing of SGM youth [13, 14], it is reasonable to suspect that SGM young people in LMICs are also experiencing the negative impacts of unsupportive environments on their physical and mental health. However, to what extent remains unknown due to a dearth of research [15].

To date, only one scoping review has explored survey research with gender minority adolescents in LMICs [16], but no scoping or systematic reviews have explored the full body of research on SGM young people in LMICs. The objective of this scoping review is to address this critical gap by reviewing literature published in the last decade with SGM young people (ages 10–24 as defined by the World Health Organization [17]) living in LMICs, that focuses on the health, safety, or wellbeing of SGM young people, and/or attitudes toward them. The primary research question this review aims to answer is: What is known about the mental and physical health needs, safety, and wellbeing of SGM young people living in LMICs?

## Methods

Our scoping review design was informed by the methodological frameworks put forth by Arksey and O’Malley and the Joanna Briggs Institute [18, 19].

### Exploratory review

A preliminary search of the literature on sexual and gender minority young people living in LMICs was conducted prior to the searches performed for the scoping review. A search of Campbell Systematic Reviews, the Cochrane Library, PubMed, PROSPERO, and JBI Evidence Synthesis revealed that no reviews of this nature had been previously conducted nor were ongoing. Additional exploratory searches were conducted on two general social and behavioral science databases (PubMed, Web of Science) and one topic-specific database (LGBTQ + Source) to ensure a robust search. The exploratory review helped to inform the keyword list for our search strategy, refine the research question, and develop the inclusion criteria and objectives.

### Study selection criteria

Peer-reviewed papers were considered eligible for inclusion in the scoping review if they:

1. Were published in English;
2. Were published between January 1, 2010, and December 31, 2020;
3. Were an empirical study (any design or methodology) or a review paper published in a peer-reviewed journal;
4. Were conducted in full or part in any low- or lower-middle- income country according to the 2021 World Bank Classification [20];
5. Included a focus on SGM populations OR the focus was on attitudes toward SGM populations;
6. Explicitly focused on any subset of youth, adolescent, young adult, or student populations aged 10–24 OR the mean of the study sample was 9.5–24.4 OR the median of the study sample was between 10–24 OR 51% of the study sample was between 10–24 OR data for a relevant outcome (see criteria #7) was disaggregated for any subset of the study sample age 10–24 OR the study retrospectively surveyed adult participants about their experiences during youth or adolescence;
7. Reported on factors influencing the health and wellbeing of SGM populations, including physical and mental health, healthcare-seeking behaviors, substance use, experiences of discrimination and/or stigma, experiences of violence and abuse (emotional, physical, and/or sexual), economically motivated paid sex practices, housing or economic security, and attitudes of others toward SGM populations

Of note, we aimed to summarize the factors influencing the health of SGM adolescents beyond their risk for HIV/AIDS, their HIV/AIDS positive status, or engagement with HIV/AIDS testing and treatment services. This body of work has been reviewed elsewhere, with reviews speaking to the emerging sexual health needs of young men who have sex with men (YMSM) [21]. While this body of work offers important insights into the health and wellness needs of SGM youth, we sought to focus on themes and outcomes that are often overlooked in the literature on SGM youth. Therefore, we excluded papers with health outcomes exclusively linked to HIV (e.g., HIV/AIDS or STI prevalence, transmission, testing, risk factors, and/or treatment).

In addition, we excluded the following types of studies and publications:

- Studies that focused on clinical outcomes related to biomedical or medical interventions (e.g., gender-affirming care)
- Conference abstracts
- Diagnostic studies (e.g., assessing the sensitivity and specificity of screening tests such as for HIV and/or STIs)
- Non-empirical studies (e.g., commentaries, editorials)
- Scale development and validation (e.g., internalized homophobia scale, attitudes toward homosexuality scale)
- Grey literature
- Protocols

### Search methods for identifying studies

In collaboration with an Information Specialist, we developed the search strategy (Appendix) for PubMed and modified it for use in the other databases. Search terms for identifying countries in the Majority World and classified by the World Bank as “low-, lower-middle-, and upper-middle- income countries” were adapted from the *EPOC LMIC filters* developed by the WHO Library and Campbell Collaboration [22].

Initial searches for articles published between January 1^st^, 2010 – December 31^st^, 2020 were conducted in February 2021 using PubMed, Web of Science, and LGBTQ + Source. An additional search was conducted in May 2023 to capture newer publications from low- and lower- middle income countries only released between January 1^st^, 2021 – May 21^st^, 2023.

Using the inclusion criteria described above, title and abstract screening were conducted in Covidence by researchers JD and CG. Full text review of for each publication was conducted by at least two of the three reviewers (JD, CF, CG). Any disputes regarding eligibility were assessed by the reviewer who had not screened the given article for inclusion at the full text stage and were resolved via discussion between all three reviewers. We imported all references into Zotero for citation management.

### Data extraction

Three reviewers independently extracted relevant data from eligible studies in Covidence. We extracted data on study characteristics, thematic focus, and study population. The extracted data was imported into Airtable for cleaning and analysis. The dataset generated during the study can be accessed here.

## Results

### Search results

**Fig. 1:**
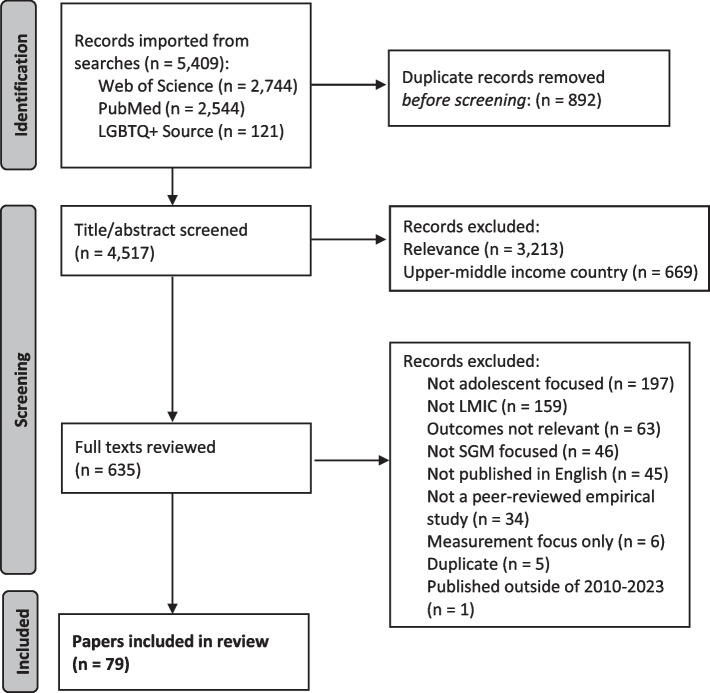
PRISMA Diagram

The PRISMA flow diagram describes the study selection process (Fig. 1). We identified 5,409 records across the three electronic databases. After removing 892 duplicate results, 4,517 unique records remained and were screened for relevance based on a review of titles and abstracts; 3,213 abstracts were excluded based on relevance. The search was designed to return records from low, lower-middle, and upper-middle income countries, however the intent of the current review is to focus on the most resource-constrained environments, therefore records from upper-middle-income countries (669) were set aside for a separate review, leaving 635 records for full text review. Articles were most commonly excluded at the full-text review stage because they focused on adult instead of youth populations (*n* = 197) or took place in a high income country (*n* = 159). Ultimately, 79 papers representing 74 unique studies were included in this review (see Table 1). Of these, 50 papers included SGM young people ages 10–24 as participants to some extent, and only 13 focused exclusively on SGM young people ages 10–24.

**Table 1:** Study Characteristics

Theme	Author (Year)	Title	Countries	Study Design	SGM Study Participants
Risks to the Health of SGM Populations	Achar & Gopal (2023) [56]	Coming out of the desi closet: disclosure of same-sex sexuality in metropolitan-India	India	Qualitative	Yes, explicitly recruited
	Agardh et al. (2016) [86]^a^	Health Risks in Same-Sex Attracted Ugandan University Students: Evidence from Two Cross-Sectional Studies	Uganda	Cross-sectional	Identified within dataset
	Alibudbud (2023a) [58]	"Does Sexual Orientation Matter?": A Comparative Analysis of the Prevalence and Determinants of Depression and Anxiety Among Heterosexual and Non-Heterosexual College Students in a University in Metro Manila	Philippines	Cross-sectional	Identified within dataset
	Alibudbud (2023b) [59]	Gender in mental health: Relationship of spirituality, social support, and COVID-19-related fear among heterosexual and LGBTQ + youth	Philippines	Cross-sectional	Identified within dataset
	Amatullah et al. (2020) [32]	Exploring identity, culture, and psychosis in cannabis dependence—an interpretative phenomenological case study from India	India	Case study	Yes, explicitly recruited
	Anderson et al. (2022) [39]	Childhood maltreatment class and sexually violent behavior among university men in Vietnam	Vietnam	Experimental	Identified within dataset
	Armstrong et al. (2020) [28]	Mental health, coping and resilience among young men who have sex with men in Zambia	Zambia	Mixed methods	Yes, explicitly recruited
	Chakrapani et al. (2022) [40]	Affirming and negotiating gender in family and social spaces: Stigma, mental health and resilience among transmasculine people in India	India	Qualitative	Yes, explicitly recruited
	Cheng et al. (2016) [75]	Same-Sex Behavior and Health Indicators of Sexually Experienced Filipino Young Adults	Philippines	Cross-sectional	Identified within dataset
	Chiongbian et al. (2023) [60]	Finding God Alongside Trials: Catholicism and Resilience Among Queer Filipino Emerging Adults	Philippines	Qualitative	Yes, explicitly recruited
	Clatts et al. (2016) [84]	Sexually transmissible infection and HIV prevention and treatment for young male sex workers in Vietnam: findings from the SHEATH intervention	Vietnam	Pretest–posttest	Yes, explicitly recruited
	Cleofas & Alibudbud (2023) [61]	Emerging From a Two-Year-Long Quarantine: A Retrospective Study on Life Satisfaction Trajectory and Depression Among Young LGBTQ + Students in the Philippines	Philippines	Cross-sectional	Yes, explicitly recruited
	Dayrit & Alibudbud (2023) [62]	The unbearable struggle for beauty: Physical appearance perfectionism, mental health, and discrimination among heterosexual cisgender and sexually diverse youth in the Philippines	Philippines	Cross-sectional	Identified within dataset
	Delany-Moretlwe et al. (2015) [100]	Providing comprehensive health services for young key populations: needs, barriers and gaps	Global	Literature review	n/a
	Folayan et al. (2022a) [43]^b^	Associations between mental health and HIV status among sexual minority and heterosexual adolescents in Nigeria	Nigeria	Cross-sectional	Identified within dataset
	Folayan et al. (2022b) [44]^b^	Associations between resilience, self-esteem, HIV status, and sexual identity among residents in Nigeria	Nigeria	Cross-sectional	Identified within dataset
	Gacusan et al. (2021) [35]	Sexual identity management of GLB emerging adults in social support contexts	Philippines	Cross-sectional	Yes, explicitly recruited
	Ganbaatar et al. (2022) [45]	Exploring the identities and experiences of young queer people in Mongolia using visual research methods	Mongolia	Qualitative	Yes, explicitly recruited
	Gaur et al. (2023) [65]	Mental healthcare for young and adolescent LGBTQ + individuals in the Indian subcontinent	India	Literature review	n/a
	Geibel et al. (2017) [96]	Stigma Reduction Training Improves Healthcare Provider Attitudes Toward, and Experiences of, Young Marginalized People in Bangladesh	Bangladesh	Pretest–posttest	Identified within dataset
	Harper et al. (2015) [87]	Resilience among gay/bisexual young men in Western Kenya: psychosocial and sexual health outcomes	Kenya	Cross-sectional	Yes, explicitly recruited
	Htut et al. (2018) [83]	Young key affected population in Myanmar: are there any challenges in seeking information and care for HIV/sexually transmitted infections and reproductive health?	Myanmar	Mixed methods	Yes, explicitly recruited
	Johansson et al. (2022) [47]	Gender non-binary adolescents' somatic and mental health throughout 2020	Morocco, Serbia, Sweden, US, Vietnam	Cross-sectional	Identified within dataset
	Johnston et al. (2017) [76]	Correlates of Forced Sex Among Young Men Who Have Sex With Men in Yangon and Monywa, Myanmar	Myanmar	Cross-sectional	Yes, explicitly recruited
	Larsson et al. (2016) [88]^a^	Determinants of unmet needs for healthcare and sexual health counselling among Ugandan university students with same-sex sexuality experience	Uganda	Cross-sectional	Identified within dataset
	Lian et al. (2015) [80]^c^	Sexual orientation and risk factors for suicidal ideation and suicide attempts: a multi-centre cross-sectional study in three Asian cities	Vietnam, China, Taiwan	Cross-sectional	Identified within dataset
	Manalastas (2016) [81]	Suicide Ideation and Suicide Attempt Among Young Lesbian and Bisexual Filipina Women: Evidence for Disparities in the Philippines	Philippines	Cross-sectional	Identified within dataset
	Mwaniki et al. (2022) [50]	"My Friends Would Believe My Word": Appropriateness and Acceptability of Respondent-Driven Sampling in Recruiting Young Tertiary Student Men Who Have Sex with Men for HIV/STI Research in Nairobi, Kenya	Kenya	Qualitative	Yes, explicitly recruited
	Newman et al. (2022a) [51]	Peer education interventions for HIV prevention and sexual health with young people in Mekong Region countries: a scoping review and conceptual framework	Cambodia, Laos, Myanmar, Thailand, Vietnam	Scoping review	n/a
	Newman et al. (2022b) [52]	HIV and mental health among young people in low-resource contexts in Southeast Asia: A qualitative investigation	Indonesia, Philippines, Thailand, Vietnam	Qualitative	Yes, explicitly recruited
	Nguyen & Angelique (2017) [72]	Internalized Homonegativity, Confucianism, and Self-Esteem at the Emergence of an LGBTQ Identity in Modern Vietnam	Vietnam	Cross-sectional	Yes, explicitly recruited
	Nowshin et al. (2022) [55]	Sexual and reproductive health and rights of "last mile" adolescents: a scoping review	Global	Scoping review	n/a
	Pham et al. (2023) [67]	Screening for adverse childhood experiences among young people using drugs in Vietnam: related factors and clinical implications	Vietnam	Cross-sectional	Yes, explicitly recruited
	Pike et al. (2023) [16]	A scoping review of survey research with gender minority adolescents and youth in low and middle-income countries	Global	Scoping review	n/a
	Quarshie et al. (2020) [26]	Prevalence of self-harm among lesbian, gay, bisexual, and transgender adolescents: a comparison of personal and social adversity with a heterosexual sample in Ghana	Ghana	Cross-sectional	Identified within dataset
	Ramadhani et al. (2020) [27]	Association of age with healthcare needs and engagement among Nigerian men who have sex with men and transgender women: cross-sectional and longitudinal analyses from an observational cohort	Nigeria	Cross-sectional	Yes, explicitly recruited
	Reyes et al. (2023) [68]	In/Out of the Closet: Perceived Social Support and Outness Among LGB Youth	Philippines	Mixed methods	Yes, explicitly recruited
	Robert et al. (2020) [30]	Factors influencing access of HIV and sexual and reproductive health services among adolescent key populations in Kenya	Kenya	Qualitative	Yes, explicitly recruited
	Sabin et al. (2018) [92]	"Too Much Sex and Alcohol": Beliefs, Attitudes, and Behaviors of Male Adolescents and Young Men Who have Sex with Men in Ghana	Ghana	Qualitative	Yes, explicitly recruited
	Yu et al. (2016) [77]	Sexual Initiation and Complex Recent Polydrug Use Patterns Among Male Sex Workers in Vietnam: A Preliminary Epidemiological Trajectory	Vietnam	Cross-sectional	Yes, explicitly recruited
Attitudes Toward SGM Populations	Adeyemo (2020) [23]	Filipino University Students' Attitude Toward Sexual Minorities: Implications for International Students	Philippines	Cross-sectional	No
	Afe et al. (2019) [85]	Social distancing toward gays and lesbians among college students in Lagos, Nigeria	Nigeria	Cross-sectional	No
	Ahuja et al. (2019) [74]	Subverting Heteronormativity: An Intervention to Foster Positive Attitudes Toward Homosexuality Among Indian College Students	India	Experimental	No
	Ardman et al. (2020) [24]	Attitudes and Knowledge of Medical Students in Hanoi regarding Lesbian and Gay People	Vietnam	Cross-sectional	Identified within dataset
	Banwari et al. (2015) [97]	Medical students and interns' knowledge about and attitude towards homosexuality	India	Cross-sectional	Identified within dataset
	Dabra & Prasad (2021) [34]	A Gap Analysis of the Perception of College Teachers and Students towards the LGBT Community	India	Cross-sectional	No
	De Costa et al. (2022) [42]	Teaching and Learning of Queer Representation in Sri Lankan English Fiction: A Reception Study within Higher Education Institutions of Sri Lanka	Sri Lanka	Qualitative	No
	Elischberger et al. (2018) [101]	Attitudes Toward and Beliefs about Transgender Youth: A Cross-Cultural Comparison Between the United States and India	India, US	Cross-sectional	Identified within dataset
	Feng et al. (2012) [79]^c^	Adolescents' and young adults' perception of homosexuality and related factors in three Asian cities	Vietnam, China, Taiwan	Cross-sectional	Identified within dataset
	Fenn et al. (2023) [64]	Awareness and attitude of medical personnel in Kerala, India to transgender persons	India	Cross-sectional	No
	Gyasi-Gyamerah et al. (2019) [95]	Changing Attitudes toward Homosexuality in Ghana: The Power of Attributional Discourse	Ghana	Pretest–posttest	No
	Kamya & White (2022) [48]	Providing Services to Youth Involved in Transactional Sex in Uganda: Professional Ethics in the Context of LGBTQ plus and Gender Oppression	Uganda	Qualitative	No
	Kar et al. (2022) [49]	Decriminalization and medical students attitudes to same sex behaviour	India	Cross-sectional	Identified within dataset
	Lowe et al. (2021) [36]	Anti-gay "Honor" Abuse: A Multinational Attitudinal Study of Collectivist- Versus Individualist-Orientated Populations in Asia and England	England, India, Iran, Malaysia, Pakistan	Cross-sectional	No
	Martins et al. (2020) [25]	The Need for Transgender Healthcare Medical Education in a Developing Country	Pakistan	Cross-sectional	No
	Mucherah et al. (2016) [89]	Grappling with the issue of homosexuality: perceptions, attitudes, and beliefs among high school students in Kenya	Kenya	Cross-sectional	No
	Nguyen & Blum (2014) [82]	Homosexuality tolerance among male and female Vietnamese youth: an examination of traditional sexual values, self-esteem, and demographic/contextual characteristics	Vietnam	Cross-sectional	No
	Nisha et al. (2022) [53]	Development of professional attitude towards dental treatment towards transgender	India	Cross-sectional	No
	Omodara & Idowu (2020) [29]	The perceptions and attitudes of undergraduate students in Obafemi Awolowo University towards Alternative Sexual Relationships (ASRs)	Nigeria	Mixed methods	No
	Rani & Samuel (2019) [98]	Reducing transphobia: comparing the efficacy of direct and indirect contact	India	Pretest–posttest	No
	Sekoni et al. (2016) [90]	Provision of Healthcare Services to Men Who Have Sex with Men in Nigeria: Students' Attitudes Following the Passage of the Same-Sex Marriage Prohibition Law	Nigeria	Cross-sectional	No
	Shah et al. (2018) [99]	First-year medical students' attitudes towards sexuality	India	Cross-sectional	No
	Tran-Thanh (2020) [33]	Queer identity inclusion in the EFL classroom: Vietnamese teachers' perspectives	Vietnam	Qualitative	No
	Winskell & Sabben (2016) [93]^d^	Sexual stigma and symbolic violence experienced, enacted, and counteracted in young Africans' writing about same-sex attraction	Benin, Burkina Faso, Cameroon, DRC, Eswatini, Kenya, Mali, Nigeria, Rwanda, Senegal	Qualitative	No
	Winskell et al. (2017) [71]^d^	Young Africans' representations of the origins of same-sex attraction and implications for sexual and mental health	Benin, Burkina Faso, Cameroon, DRC, Eswatini, Kenya, Mali, Nigeria, Rwanda, Senegal	Qualitative	No
	Winskell et al. (2018) [94]^d^	From condemnation to normalisation: Young Africans' narratives about same-sex attraction and implications for communication and advocacy efforts	Benin, Burkina Faso, Cameroon, DRC, Eswatini, Kenya, Mali, Nigeria, Rwanda, Senegal	Qualitative	No
Experiences of Stigma and Discrimination among SGM Populations	Alam & Marston (2023) [57]	'Bending' against straightening devices: queer lived experiences of sexuality and sexual health in Bangladesh	Bangladesh	Qualitative	Yes, explicitly recruited
	Alizai et al. (2017) [73]	Impact of Gender Binarism on Hijras' Life Course and Their Access to Fundamental Human Rights in Pakistan	Pakistan	Qualitative	Yes, explicitly recruited
	Coleman et al. (2018) [78]	Gender Variance and Sexual Orientation Among Male Spirit Mediums in Myanmar	Myanmar	Qualitative	Yes, explicitly recruited
	Crankshaw et al. (2022) [41]	Intersectional vulnerabilities and differential impacts of COVID-19 responses on young people who sell sex in Zimbabwe	Zimbabwe	Qualitative	Yes, explicitly recruited
	de Lind van Wijngaarden et al. (2013) [69]	Sexual abuse, social stigma and HIV vulnerability among young feminised men in Lahore and Karachi, Pakistan	Pakistan	Qualitative	Yes, explicitly recruited
	Embleton et al. (2023) [63]	Intersectional Stigma and Implementation of HIV Prevention and Treatment Services for Adolescents Living with and at Risk for HIV: Opportunities for Improvement in the HIV Continuum in Sub‑Saharan Africa	Kenya, Ghana, Uganda	Multiple case study	n/a
	Horton (2014) [70]	'I thought I was the only one': the misrecognition of LGBT youth in contemporary Vietnam	Vietnam	Qualitative	Yes, explicitly recruited
	Huynh et al. (2022) [46]	Aggression Toward LGBT Students and the Role of Social Workers in Vietnam	Vietnam	Mixed methods	Yes, explicitly recruited
	Meer & Muller (2023) [66]	Making 'meanwhile …': representing queer African youth through spontaneous collaborative graphic autoethnography	Botswana, Kenya, Zimbabwe	Qualitative	Yes, explicitly recruited
	Noor et al. (2021) [37]	Resourcefulness of homeless young people who practise sex work in Pakistan: a qualitative study	Pakistan	Qualitative	Identified within dataset
	Noor (2022) [54]	Violence against homeless gay and transgender youth in Pakistan – a short report	Pakistan	Qualitative	Yes, explicitly recruited
	Okanlawon (2020) [31]	Homophobia in Nigerian schools and universities: Victimization, Mental Health Issues, Resilience of the LGBT Students and support from Straight Allies. A Literature review	Nigeria	Literature review	n/a
	Quarshie (2021) [38]	Boys should not be overlooked: Sexual violence victimization and associated factors among school-going adolescents in urban Ghana	Ghana	Cross-sectional	Identified within dataset

^a-d^denote publications that derive from the same study

### Study characteristics

The 79 included papers were published between January 2010 and May 2023 across 56 unique peer-reviewed journals. The majority of papers included in our review were published between 2020–2023 (*n* = 47, 59.49%) [16, 23–68]. There were 47 papers published between 2020–2023, which is more than the number of papers published during the preceding decade. The journals with the most publications were Culture, Health & Sexuality (*n* = 7, 8.86%) [28, 40, 41, 45, 69–71], Journal of Homosexuality (*n* = 5, 6.33%) [24, 58, 72–74], and Archives of Sexual Behavior (*n* = 4, 5.06%), [75–78].

**Fig. 2:**
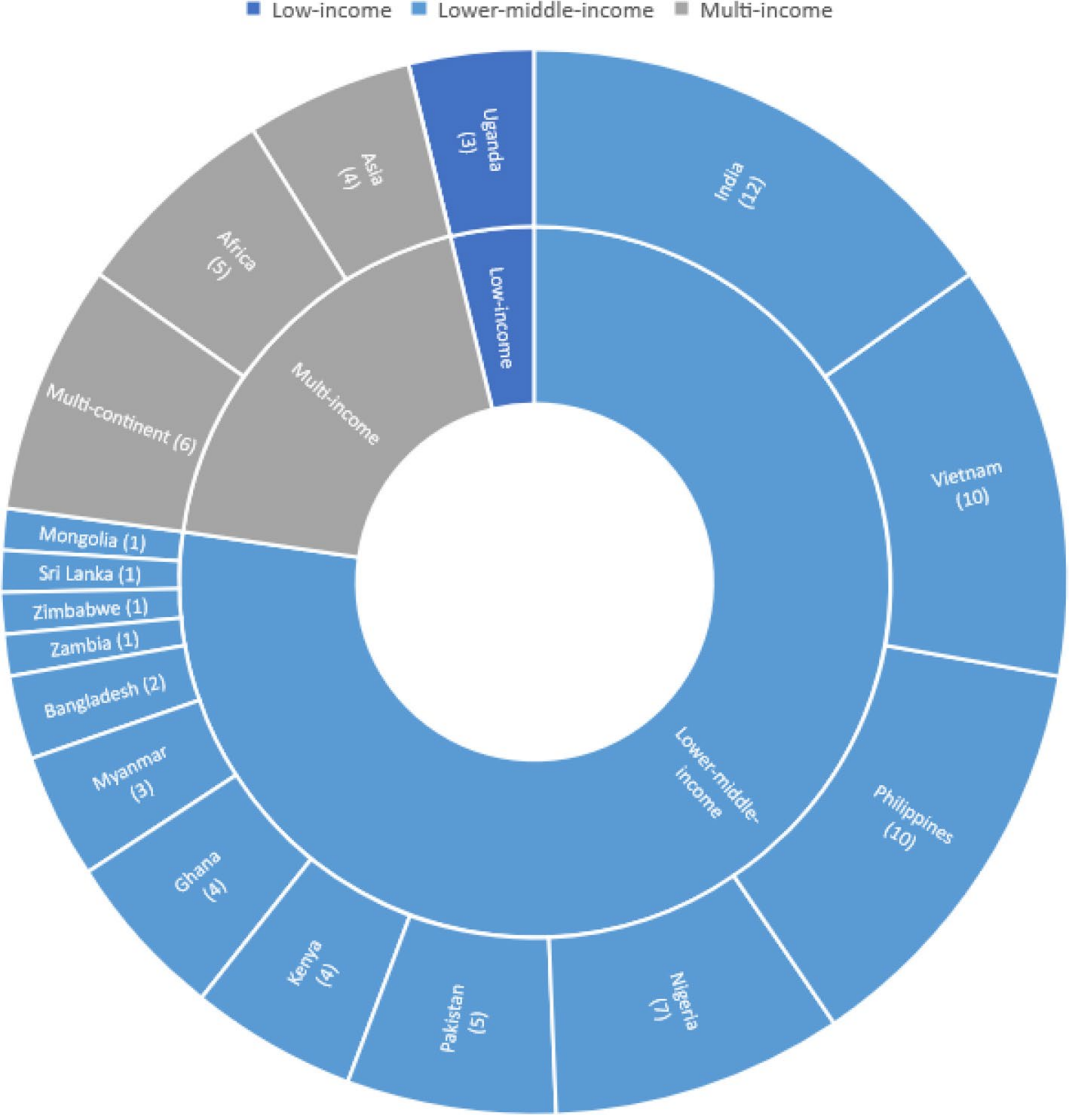
Included Papers by Country and Income Classification

The 79 included papers drew primarily from data collected in East Asia and the Pacific (*n* = 28, 35.44%) [23, 24, 33, 35, 39, 45, 46, 51, 52, 58–62, 68, 70, 72, 75–84], Sub-Saharan Africa (*n* = 25, 31.65%) [26–31, 38, 41, 43, 44, 50, 63, 66, 71, 85–95], and South Asia (*n* = 20, 25.32%) [25, 32, 34, 40, 42, 49, 53, 54, 54, 56, 57, 64, 65, 69, 73, 74, 96–99] (see Fig. 2). There were no papers identified from low- and lower-middle- income countries in the Middle East and North Africa or Latin America and the Caribbean.

Only three publications drew from studies conducted in low-income countries, all in Uganda [48, 86, 88]. Seventy-three papers described studies conducted, at least in part, in lower-middle-income countries, with the most from India (*n* = 12, 15.19%) [32, 34, 40, 49, 53, 56, 64, 65, 74, 97–99], the Philippines (*n* = 10, 12.66%) [23, 35, 58–62, 68, 75, 81], and Vietnam (*n* = 10, 12.66%) [24, 33, 39, 46, 67, 70, 72, 77, 82, 84]. Three papers were global in scope [16, 55, 100].

Almost half of the papers (*n* = 37, 46.84%) were cross-sectional studies [23–27, 34–36, 38, 43, 47, 49, 53, 58, 59, 61, 62, 64, 67, 72, 75–77, 79–82, 85–91, 97, 99, 101] (see Table 1). An additional twenty-three publications (29.11%) employed qualitative designs [30, 33, 37, 40–42, 45, 48, 50, 52, 54, 56, 57, 60, 66, 69–71, 73, 78, 92–94], five (6.33%) were mixed methods [28, 29, 46, 68, 83], four used a pre-post design (5.06%) [84, 95, 96, 98], and two (2.53%) were case studies [32, 63]. Only two of the 79 included papers (2.53%) were experimental and included a randomized intervention and control group [39, 74]. The remaining six papers (7.60%) were reviews (scoping and literature) that included and/or focused on SGM young people [16, 31, 51, 55, 65, 100]. There were no longitudinal studies.

Less than half of the papers (*n* = 35, 44.30%) exclusively recruited or focused on young people ages 10–24 or any subset of the 10–24 age range [26, 28, 30, 35, 38, 39, 41, 43, 45–47, 50, 52, 57, 58, 60, 61, 66–69, 71, 74, 75, 79–83, 89, 93, 94, 96, 98, 99]. Twenty-six additional papers (32.91%) had a sample that was made up of majority young people (as determined by mean, median, or majority criteria) [23–25, 27, 29, 36, 44, 49, 53, 56, 59, 62, 64, 70, 72, 76, 77, 84–88, 90, 92, 95, 97]. Within these twenty-six papers, age-disaggregated data for any subset of the 10–24 adolescent age band was reported in only five (6.33%) and the reported age groupings varied [27, 53, 77, 85, 92]. Nine papers (11.39%) focused on adults. Of these, four (5.063%) were retrospective accounts of adolescence [32, 40, 73, 78] and five (6.33%) surveyed adults on their attitudes toward SGM young people [33, 34, 42, 48, 101]. We also included six review papers (7.59%) that identified SGM adolescents as one of their populations of interest [16, 31, 51, 55, 65, 100].

Almost two-thirds of included papers (*n* = 50, 63.29%) included SGM individuals—at least to some extent—as participants. In 30 papers (37.97%), SGM populations were specifically recruited as study participants [27, 28, 30, 32, 35, 40, 41, 45, 46, 50, 52, 54, 56, 57, 60, 61, 66–70, 72, 73, 76–78, 83, 84, 87, 92] and the remaining 20 (25.31%) studied a broader population but provided disaggregated data on SGM study participants [24, 26, 37–39, 43, 44, 47, 49, 58, 59, 62, 75, 79–81, 86, 88, 96, 97]. Moreover, when taking into account age of study participants, only 27 of the 50 papers with SGM study participants focused exclusively on young people (ages 10–24) [26, 28, 30, 35, 37–39, 41, 43, 45–47, 50, 52, 54, 57, 58, 60, 61, 66–69, 75, 79–81, 83, 96].

Lastly, over half of the papers in this review (*n* = 41, 51.90%) drew from data that was collected in LMICs that criminalized same-sex relations and/or forms of gender expression at the time the study was conducted [25–30, 32, 36–38, 41–44, 48, 50, 54, 57, 66, 69, 71, 73, 74, 76, 78, 83, 85–90, 92–99, 101]. While many of these 41 studies focused on attitudes towards SGM populations rather than SGM populations themselves, it is important to highlight that SGM young people were specifically recruited and participated in 14 (17.72%) of these studies [27, 28, 30, 41, 54, 57, 66, 69, 73, 76, 83, 87, 91, 92]. Nine (11.39%) studies successfully recruited SGM young people under age 18 in the study sample [27, 30, 41, 54, 69, 73, 76, 83, 92].

### Key findings by theme

The included papers (79) were classified according to their thematic focus. Across the review, three key groupings emerged:

1. Risks to the health of SGM populations (*n* = 40)
2. Attitudes toward SGM populations (*n* = 26)
3. Experiences of stigma and discrimination among SGM populations (*n* = 13)

#### Risks to the health of SGM populations

Half of the included papers (*n* = 40, 50.63%) examined the mental health, sexual health, and resilience of SGM young people, as well as comparative studies that explored the differential health risks that SGM young people are subjected to compared to their cisgender and/or heterosexual peers. [16, 26–28, 30, 32, 35, 39, 40, 43–45, 47, 50–52, 55, 56, 58–62, 65, 67, 68, 72, 75–77, 80, 81, 83, 84, 86–88, 92, 96, 100]. Additionally, three scoping reviews [16, 51, 55] and two literature reviews were included in this category [65, 100], such as Pike et al.’s assessment of existing survey research on gender minority adolescents in low- and middle- income countries [16].

##### Mental health

Twenty-three papers (*n* = 23, 29.11%) explored different aspects of the mental health and emotional wellbeing of SGM young people [26, 28, 32, 35, 39, 40, 43–45, 47, 56, 58–62, 67, 68, 70, 72, 80, 81, 87], of which fourteen focused exclusively on SGM participants [28, 32, 35, 40, 45, 47, 56, 60, 61, 67, 68, 70, 72, 87]. These 14 papers covered topics such as SGM identity management and the need to conceal SGM identities in many social contexts to feel safe, the impact of religion on internalized homo/transphobia, and experiences of violence. The remaining nine papers were comparative studies that investigated the prevalence of adverse mental health outcomes (i.e., anxiety, depression, and self-harm) and protective factors (i.e., social support) of SGM young people compared to their cisgender and/or heterosexual peers [26, 39, 43, 44, 58, 59, 62, 80, 81]. Three of the nine comparative studies explored the mental health of SGM youth in the Philippines, and found that SGM youth were at increased risk for anxiety, depression, appearance perfectionism, and suicidal ideation compared to their cisgender and/or heterosexual peers [58, 62, 81]. Similarly, a study of Nigerian adolescents (with two publications that explored the same dataset) found that sexual minority individuals reported lower resilience and self-esteem when compared to their heterosexual counterparts [43, 44]. Quarshie et al. presented only descriptive statistics but also found that Ghanaian SGM youth also had worse mental health outcomes than their non-SGM peers [26]. In contrast, Lian et al. found that higher risk of suicidal ideation among sexual minority adolescents was location-specific and only seen in one of the three southeast Asian cities studied [80].

##### Sexual health

Twelve papers (15.19%) focused on aspects of SGM sexual health, of which nearly all (*n* = 7) focused on YMSM and/or transgender people (*n* = 2) within a key populations HIV framework^2^rather than SGM young people explicitly [27, 30, 50, 52, 77, 83, 84, 92, 96]. For example, one study was an evaluation of a harm reduction and sexual health promotion intervention for young male sex workers who have sex with men in Vietnam [84], while another was an assessment of client satisfaction with sexual and reproductive health services among adolescent key populations in Bangladesh [96]. Also included in this grouping is a formative study exploring the acceptability of respondent-driven sampling strategies for recruitment of YMSM [50]. The remaining three publications explored both the sexual health and mental health needs of SGM young people using a broader SGM framework. For example, Larsson et al. and Agradh et al.’s publications (drawing from the same dataset) used both self-reported measures of sexual attraction and sexual behavior to define and explore the unmet health needs of university students in Uganda [88]. Cheng et al. similarly used measures of same-sex sexual behavior and same-sex romantic experience to explore the mental and sexual health outcomes of Filipino young people [75].

#### Attitudes toward SGM populations

Nearly one-third (*n* = 26, 32.91%) of all included papers focused on attitudes toward SGM populations. The study participants included college or university students [23, 29, 34, 36, 42, 74, 85, 95, 98], high school students [89], health professions students [24, 25, 49, 53, 90, 97, 99], adolescents [71, 79, 82, 93, 94], and non-SGM adults [33, 48, 101]. Of these twenty-six papers, only four had SGM study participants [24, 49, 79, 97] and none explicitly discussed how the attitudes of others directly impact the experiences of SGM young people themselves. Three papers assessed interventions that sought to change harmful attitudes among college students toward SGM populations [74, 95, 98], although only one employed a randomized control study design [74]. Collectively, these twenty-six studies reported prevailing prejudiced, discriminatory, and harmful attitudes toward SGM communities.

#### Experiences of stigma and discrimination among SGM populations

The thirteen papers (16.46%) under this theme focused on SGM young peoples’ experiences of identity-based discrimination throughout the life course, and the impact of living in a society rife with homophobia, transphobia, and the social construct of gender binarism. Nine papers in this category, including a review, focused on gender minority and/or sexual minority populations only [31, 46, 57, 63, 66, 69, 70, 73, 78] and one explored the experiences of SGM young people in a larger study of school-going adolescents [38]. Additionally, three studies focused on young SGM people who sell sex [41] and/or are homeless [37, 54]. Collectively, these thirteen studies explored discrimination in the context of a wide variety of life experiences, from emotional and sexual to educational and occupational.

Internalized homophobia and transphobia experienced by SGM young people, and the role that inter- and intra- personal factors play in negatively impacting mental and physical health outcomes were explored in four qualitative studies [31, 69, 73, 78]. These studies highlight the struggles SGM young people face worldwide as they grow up in a global society that emphasizes heteronormativity and cisnormativity. These studies documented a range of harmful experiences, including bullying and lack of support at school [31] and violent treatment by police [69, 73].

## Discussion

To the best of our knowledge, this is the first review, scoping, systematic, or otherwise, to explore what is known about the mental and physical health needs, safety, and wellbeing of SGM young people living in low- and lower-middle- income countries. Our review shows that there is a lack of critical evidence about the mental and physical health needs, safety, and wellbeing of SGM young people in LMICs. The findings indicate that the research that does exist for SGM young people in LMICs is often about SGM young people, rather than with and for them. While research on SGM young people in recent years appears to be growing, of the 79 papers included in this review, only one-third (*n* = 29, 34.18%) specifically focused on SGM young people ages 10–24 [26, 28, 30, 35, 38, 39, 41, 43, 45–47, 50, 52, 57, 58, 60, 61, 66–69, 75, 79–81, 83, 96]. Research that focused on young sexual minority women or women who have sex with women (WSW) [81] and non-binary adolescents [47] in LMICs was the most lacking in this review, with just one included study each (excluding reviews). Additionally, although this review included extensive search terms for all LMICs (see Appendix)—265 country terms, 31 regional terms, and 54 LMIC text words—only three included papers utilized data from studies conducted exclusively in a low-income country (all from Uganda), with the majority of included papers drawing on data from lower-middle-income countries. Our review highlights the need for more research with and for SGM young people living in LMICs, especially in the most resource-constrained environments.

We also included papers that focused on attitudes toward SGM populations. These made up nearly one-third of all of the papers (*n* = 26, 32.91%) included in this review [23–25, 29, 33, 34, 36, 42, 48, 49, 53, 64, 71, 74, 79, 82, 85, 89, 90, 93–95, 97–99, 101]. The findings from these papers make it clear that discriminatory and hostile homophobic and transphobic attitudes towards SGM young people remain prevalent. While studies focused on attitudes can offer insight into how SGM young people are perceived in different contexts and settings, these studies do not provide information on how these harmful attitudes impact the physical and mental health of SGM young people. Thus, there is a critical need for studies that center the experience of SGM young people themselves. Only thirteen papers explored the differential health risks that SGM young people are subjected to compared to their cisgender and/or heterosexual peers. The limited number of studies identified and their differing findings suggest that more research is needed in order to better understand which adverse health outcomes SGM young people are at a higher risk of experiencing and in which settings [26, 26, 39, 43, 43, 59, 59, 62, 75, 80, 81, 86, 88].

### Perpetuating erasure

Throughout the review, data on participants’ sexual and gender identity was limited and inconsistently reported. In order to better understand the needs and experiences of sexual and gender minority young people, demographic data about gender and sexual identity must be more accurately captured and regularly reported [16, 103]. In addition, there were many studies in this review that set out to recruit young adult populations but defined young people with a larger age band than that set by this review. Despite these larger age bands, age-disaggregated data was only reported in five of these papers [27, 77, 85, 91, 92]. The lack of age-disaggregated data erases the experiences of SGM youth populations. Age data should be broken out in developmentally appropriate age bands, such as 10–14 for young adolescents, 15–19 for older adolescents, and 20–24 for young adults [104]. This is critical for SGM young people (and for all young people in general) to become visible in the data, so their mental and physical health needs can be better understood and met with evidence-based services and programs, and included in global initiatives such as the United Nation’s 2030 Agenda and Sustainable Development Goals (SDGs) [105].

That said, we recognize that sample size limitations can preclude age-disaggregation. For example, Johnston et al.’s study was originally focused on recruiting young people ages 15–24 and they report having to increase the age limit to 28 to boost recruitment [76]. Additionally, it may not be possible to disaggregate datasets due to the potential risk of individuals becoming identifiable in the data [104]. Ramadhani et al. noted that a limitation of their study was the small sample of transgender women participants, ultimately leading to the authors’ decision to pool transgender women and MSM survey respondents [27].

### A way forward

There is no one-size-fits-all solution to increasing participation of SGM young people in research and safely capturing more inclusive demographic data. However, two strategies adopted by studies included in this review that researchers could consider utilizing are community-based participatory research (CBPR) approaches and working with local youth networks to co-design research directly with SGM young people (Table 2). For example, Ganbaatar et al. and Meer and Muller, integrated CBPR principles with their qualitative study designs to effectively engage with young SGM populations and shine a light on their lived experiences [45, 66]. Ganbaatar et al. used photo-elicitation with queer young people in Mongolia to better understand how youth navigate their SGM identities, and Meer and Muller worked collaboratively with SGM youth from Zimbabwe, Kenya, and Botswana to create an anthology of graphic short stories that represented their lived experiences as queer Africans. Additionally, a study on unmet HIV and mental health needs in Southeast Asia was designed to work collaboratively with a local youth network to develop study tools relevant to the lives of adolescents and young key populations, including SGM young people [52]. Not only does meaningful involvement of the target population in research design and implementation result in more culturally appropriate survey tools, but it can also promote wellbeing and resilience among its participants [106]. Obtaining waivers of parental consent from ethical review boards is also a potential strategy that could increase participation (and potentially safety) of SGM youth in research. This is a strategy that is sometimes necessary in order to safeguard and encourage research informed by SGM young people, mitigate potential risk due to forced disclosure of sexual orientation and/or gender identity, and help to ensure that SGM young people who are at high risk for negative health outcomes are included in critical research [107, 108].

**Table 2:** Recommendations for Research with SGM Young People in LMICs

• Develop guidance on the ethics of research with young SGM people to increase participation in research
• Adopt CBPR principles and allow SGM study participants to co-develop research aims, tools, and procedures
• Partner with local youth networks, influencers, and inclusive CBOs to recruit participants and encourage buy-in
• Obtain waivers of parental consent from ethical review boards
• Conduct risk assessments and develop risk mitigation plans for ensuring the confidentiality and safety of all study participants
• Leverage online tools to recruit study participants and/or collect data

Alliance building with SGM-led community based organizations (CBOs) will also be key to continuing research with SGM young people in settings where same-sex relations between consenting adults and/or forms of gender diverse expression are criminalized [109]. This is more important now than ever given the escalating political volatility towards SGM individuals around the globe [110, 111]. This review identified 43 papers that draw from data collected in 19 different countries where SGM identities and behaviors are criminalized,^3^ demonstrating that it is possible to conduct research on sexual and gender diversity in these settings [25–30, 32, 36–38, 41–44, 47, 48, 50, 52, 54, 57, 66, 69, 71, 73, 74, 76, 78, 83, 85–90, 92–99, 101]. Importantly, SGM young people were recruited directly as study participants in 27 of these 43 papers [27, 28, 30, 32, 41, 52, 54, 57, 66, 69, 73, 76, 78, 83, 87, 91, 92].

Guidance on the ethics of research with young SGM people is needed to increase participation of SGM young people in research. When conducting research in countries with laws that are restrictive or punitive, where there is significant risk, it is paramount that risk assessments are conducted together with local SGM-led organizations and groups before, during, and after all studies so that the health and safety of participants can be protected [113]. Example tactics, as utilized in a feasibility study with adult MSM in Kenya, Malawi, and South Africa, include developing risk mitigation plans for each study site that outline protocols for ensuring the safety and confidentiality of study participants; requiring study staff and relevant stakeholders to sign confidentiality agreements; and forming advisory committees that include people from the priority population to oversee study implementation [113].

In contexts where internet is accessible, conducting virtual studies may allow for greater participant safety and anonymity [109]. There are known risks, however, with relying primarily on social media and chat groups for health information and peer support. Misinformation and cyberbullying can be pervasive, algorithmic biases exacerbate existing gender, class, education, and geographic inequalities, and sharing health information online raises privacy concerns [114]. Although researchers should be aware and take precautions against these risks, virtual tools have the potential to increase participation of SGM people who have online access. For example, a recent study with queer women and transgender men in Kenya used online sites and platforms to both recruit participants and conduct one-on-interviews. In order to promote safety of the study participants, interested participants were given an information sheet about online privacy and were asked to complete a self-assessment about potential individual risks [115]. Another study with adult SGM participants from Zimbabwe and South Africa involved responding to qualitative study questions using voice notes and/or texts over WhatsApp. The study authors found that the anonymity of participants could be maintained by having the participants send their responses to an intermediary instead of directly to the researchers [116].

### Challenges and limitations

This review is not without its own challenges and limitations. While more adolescent-focused HIV research is certainly needed, we chose to focus this review on outcomes not linked to HIV [117]. We felt that this was important to shed light on other topics related to SGM health and wellbeing, however, we understand that this has limited the scope of the review and may have left out relevant findings from the HIV-focused body of literature.

Due to time and budget constraints, we prioritized SGM adolescents living in LMICs and therefore limited our focus only to peer-reviewed research from low- and lower-middle- income countries published between 2010 and May 2023. We recognize the need for a larger global review on the health and wellbeing of SGM adolescents.

Additionally, our review was limited to three research databases and papers published in English only, and as a result, we may be missing valuable research published locally in LMICs. We also did not directly reach out to authors of the included publications; this would have provided additional insight into how some of the barriers to conducting studies in countries where same-sex relations and/or gender diverse expression are criminalized were overcome and research was safely conducted with young people. As white researchers based in the United States, we recognize that we live and work within a different cultural context than the populations we are studying. We acknowledge that this lens impacts and limits our work and our understanding of this body of literature.

## Conclusion

Our findings demonstrate a clear lack of rigorous research with SGM young people in LMICs. While this topic has received increased attention in the past four years, there is still much that is unknown about the mental and physical health needs of SGM young people in LMICs due to the limited and disjointed nature of the research that exists. We must advocate for rigorous studies with age, sexual orientation, and gender-disaggregated data that are co-designed with SGM young people and SGM-led CBOs from the outset, to not only better understand the experiences of SGM adolescents at various stages of development and across all gender and sexual identities, but also to develop solutions that more accurately identify and meet their needs. 

## Data Availability

The dataset generated during the current study is available on Airtable: https://airtable.com/shruocrdN41vNm3Y9.

## Appendix

### Search Strategy

The search strategy was used to search PubMed, LGBTQ+ Source and Web of Science in February 2021.

**Table:**

**Database Name**	**Platform**	**Web address**	**Search Field Used**
LGBTQ+ Source	EBSCO	https://www.ebsco.com/products/research-databases/lgbtq-life	Abstract; academic journals only
PubMed	NLM.NIH	https://www.ncbi.nlm.nih.gov/pubmed/advanced	Text word
Web of Science	EBSCO/Reuters	https://webofknowledge.com	Topic

The search terms were grouped by: 

- Geographic Set (S4) 
- Year Published Set (S5) 
- Sexual and Gender Minoritized Groups Set (S10)
- Beliefs and Behaviors Set (S16)
- Life Stage Set (S19)

**Table:**

**S#**	**SET NAME**	**QUERY: BOOLEAN/PHRASE**
S1	COUNTRIES	afghanistan OR albania OR algeria OR american samoa OR angola OR antigua OR barbuda OR argentina OR armenia OR armenian OR aruba OR azerbaijan OR bahrain OR bangladesh OR barbados OR belarus OR byelarus OR belorussia OR byelorussian OR belize OR british honduras OR benin OR dahomey OR bhutan OR bolivia OR bosnia OR herzegovina OR botswana OR bechuanaland OR brazil OR brasil OR bulgaria OR burkina faso OR burkina fasso OR upper volta OR burundi OR urundi OR cabo verde OR cape verde OR cambodia OR kampuchea OR khmer republic OR cameroon OR cameron OR cameroun OR central african republic OR ubangi shari OR chad OR chile OR china OR colombia OR comoros OR comoro islands OR mayotte OR congo OR zaire OR costa rica OR cote d’ivoire OR cote d’ ivoire OR cote divoire OR cote d ivoire OR ivory coast OR croatia OR cuba OR cyprus OR czech republic OR czechoslovakia OR djibouti OR french somaliland OR dominica OR dominican republic OR ecuador OR egypt OR united arab republic OR el salvador OR equatorial guinea OR spanish guinea OR eritrea OR estonia OR eswatini OR swaziland OR ethiopia OR fiji OR gabon OR gabonese republic OR gambia OR georgia OR georgian OR ghana OR gold coast OR gibraltar OR greece OR grenada OR guam OR guatemala OR guinea OR guyana OR guiana OR haiti OR hispaniola OR honduras OR hungary OR india OR indonesia OR timor OR iran OR iraq OR “isle of man” OR jamaica OR jordan OR kazakhstan OR kazakh OR kenya OR korea OR kosovo OR kyrgyzstan OR kirghizia OR kirgizstan OR kyrgyz republic OR kirghiz OR laos OR lao pdr OR lao people's democratic republic OR latvia OR lebanon OR lesotho OR basutoland OR liberia OR libya OR libyan arab jamahiriya OR lithuania OR macau OR macao OR macedonia OR madagascar OR malagasy republic OR malawi OR nyasaland OR malaysia OR maldives OR indian ocean OR mali OR malta OR micronesia OR kiribati OR marshall islands OR nauru OR northern mariana islands OR palau OR tuvalu OR mauritania OR mauritius OR mexico OR moldova OR moldovian OR mongolia OR montenegro OR morocco OR ifni OR mozambique OR portuguese east africa OR myanmar OR burma OR namibia OR nepal OR netherlands antilles OR nicaragua OR niger OR nigeria OR oman OR muscat OR pakistan OR panama OR papua new guinea OR paraguay OR peru OR philippines OR philipines OR phillipines OR phillippines OR poland OR polish people's republic OR portugal OR portuguese republic OR puerto rico OR romania OR russia OR russian federation OR ussr OR soviet union OR “union of soviet socialist republics” OR rwanda OR ruanda OR samoa OR pacific islands OR polynesia OR samoan islands OR “sao tome and principe” OR saudi arabia OR senegal OR serbia OR seychelles OR sierra leone OR slovakia OR slovak republic OR slovenia OR melanesia OR solomon island OR solomon islands OR norfolk island OR somalia OR south africa OR south sudan OR sri lanka OR ceylon OR “saint kitts and nevis” OR “st kitts and nevis” OR saint lucia OR st lucia OR saint vincent OR st vincent OR grenadines OR sudan OR suriname OR surinam OR syria OR syrian arab republic OR tajikistan OR tadjikistan OR tadzhikistan OR tadzhik OR tanzania OR tanganyika OR thailand OR siam OR timor leste OR east timor OR togo OR togolese republic OR tonga OR trinidad OR tobago OR tunisia OR turkey OR turkmenistan OR turkmen OR uganda OR ukraine OR uruguay OR uzbekistan OR uzbek OR vanuatu OR new hebrides OR venezuela OR vietnam OR viet nam OR middle east OR west bank OR gaza OR palestine OR yemen OR yugoslavia OR zambia OR zimbabwe OR northern rhodesia
S2	REGION	global south OR “africa south of the sahara” OR sub saharan africa OR subsaharan africa OR central africa OR north africa OR northern africa OR magreb OR maghrib OR sahara OR southern africa OR east africa OR eastern africa OR west africa OR western africa OR west indies OR indian ocean islands OR caribbean OR central america OR latin america OR south america OR central asia OR north asia OR northern asia OR southeastern asia OR south eastern asia OR southeast asia OR south east asia OR western asia OR east europe OR eastern europe
S3	LMIC TEXTWORDS	developing countr* OR developing nation* OR developing population* OR developing world OR less* developed countr* OR less* developed nation* OR less developed world OR under developed countr* OR under developed nation* OR under developed world OR underdeveloped countr* OR underdeveloped nation* OR underdeveloped population* OR underdeveloped world OR middle income countr* OR middle income nation* OR middle income population* OR low income countr* OR lower income countr* OR low income nation* OR lower income nation* OR low income population* OR lower income population* OR underserved countr* OR underserved nation* OR underserved population* OR under served population* OR deprived countr* OR deprived population* OR poor* countr* OR poor* nation* OR poor* population* OR poor world OR developing econom* OR less developed econom* OR underdeveloped econom* OR middle income econom* OR low income econom* OR lower income econom* OR low gdp OR lower gdp OR low gnp OR lower gnp OR low gross domestic OR lower gross domestic OR low gross national OR lower gross national OR lmic OR lmics OR third world OR lami countr* OR transitional countr* OR emerging econom* OR emerging nation*
S4	GEOGRAPHIC SET	S1 or S2 OR S3
S5	YEAR PUBLISHED	YR 2010-2020
S6	GEO/YR SET	S4 AND S5
S7	SEXUAL MINORITY	homosexual OR gay OR lesbian OR bisexual OR asexual OR LGBT* OR queer OR “same sex” OR "sexual minorit*" OR "sexual orientation" OR “sexual divers*”
S8	GENDER MINORITY	transgender OR nonbinary* OR “non binary*” OR "gender expansive" OR “gender nonconforming*” OR "gender non conforming*" OR genderqueer OR “gender queer” OR “gender fluid” OR genderfluid OR “gender minorit*” OR "gender ident*" OR “gender divers*” OR “third gender”
S9	DIFFERENCES IN SEX TRAITS OR REPRODUCTIVE ANATOMY	intersex
S10	SEXUAL AND GENDER MINORITIZED GROUPS SET	S7 OR S8 OR S9
S11	SGM/GEO/YR SET	S6 AND S10
S12	SEXUAL BEHAVIOR	“men who have sex with men” OR MSM OR “women who have sex with women” OR WSW
S13	BELIEFS & ATTITUDES	heteronormativity OR homophobia OR transphobia OR biphobia
S14	BELIEFS AND BEHAVIORS SET	S12 or S13
S15	BEL/SGM/GEO/YR SET	S11 AND S14
S16	PHASE OF LIFE	adolescence OR youth or student
S17	PERSONS BETWEEN THE AGES OF 10-24	adolescen* OR “young people” OR “young adults” OR teen OR student
S18	LIFE STAGE SET	S16 OR S17
S19	LIFE/BEL/SGM/GEO/YR SET	S15 AND S18

## Abbreviations

CBOs: Community-based organizations
CBPR: Community-based participatory research
HIV/AIDS: Human immunodeficiency virus/Acquired immunodeficiency syndrome
LGBTQI: Lesbian, gay, bisexual, transgender, queer, and intersex
LMICs: Low- and lower- middle- income countries
SDG: Sustainable Development Goals
SGM: Sexual and gender minority
STI: Sexually transmitted infection
WSW: Women who have sex with women
(Y)MSM: (Young) men who have sex with men

## Footnotes

1 In this paper, we are using the term “sexual and gender minority populations” (SGM), for people whose sex assigned at birth, sexuality, gender identity, and/or gender expression do not conform to majority heteronormative social norms, including lesbian, gay, bisexual, transgender, queer, and intersex (LGBTQI) individuals [2].

2 The Joint United Nations Program on HIV/AIDS describes key populations as groups defined by their engagement in a specific set of higher-risk behaviors that increase risk of HIV transmission and infection including YMSM, transgender women, sex workers, people who inject drugs, and incarcerated people [102].

3 This is inclusive of six papers that described studies that were conducted in India prior to the 2018 Indian Supreme Court decision that decriminalized homosexuality   [112].
